# Postpartum Bilateral Tension Pneumothoraces

**DOI:** 10.7759/cureus.4856

**Published:** 2019-06-07

**Authors:** Patrick D Collins, Samuel J McFerran, Isabelle Goldrick, Joaquim Cevallos-Morales, Juan Martin-Lazaro

**Affiliations:** 1 Intensive Care Unit, Newham University Hospital, London, GBR; 2 Intensive Care Unit, St Bartholomew's Hospital, London, GBR

**Keywords:** pneumothorax, postpartum, resuscitation, lymphangioleiomyomatosis, chest  drain, interstitial lung disease, maternal complications, ultrasound, emergency medicine, ketamine

## Abstract

We report the first case report of postpartum bilateral tension pneumothoraces. A 31-year-old primigravida presented with obstructive shock and respiratory failure five days following a normal spontaneous vaginal delivery. Bilateral surgical chest drains were inserted and following computed tomography suggestive of an underlying interstitial lung disease she was transferred to a tertiary cardiothoracic centre. Video-assisted thoracic surgery was carried out with left apicectomy and parietal pleurectomy. Histopathology supported a diagnosis of pulmonary lymphangioleiomyomatosis. We discuss the pathophysiology of labour-induced barotrauma and examine pertinent elements of the acute management of this case.

## Introduction

The decline in maternal mortality worldwide is a major human achievement. Nevertheless, in the United Kingdom the maternal mortality rate remains 9.8 per 100,000 during pregnancy and the postpartum period [1]. Improvements in perinatal care mean that the predominant causes of mortality are now related to infection or cardio-respiratory complications rather than the more classically obstetric sequelae (haemorrhage, eclampsia, obstructed labour) more prominent in the developing world [2]. Due to this changing epidemiology, there have been calls for increased awareness of the medical and surgical complications of pregnancy amongst both general physicians and clinicians involved directly in perinatal care in the United Kingdom [1]. In this report we aim to highlight a rare but potentially life-threatening complication during the postpartum period.

## Case presentation

A 31-year-old primigravida presented to a maternity unit with shoulder tip pain two days after an uncomplicated spontaneous vaginal delivery. She was born in Nigeria and moved to the United Kingdom when she was seven years old. She had a normal gestation and childhood. Her family’s medical history was unremarkable. She had a past medical history of gastro-oesophageal reflux disease and uterine fibroids. In the month prior to her presentation she had sustained a traumatic comminuted fracture of her right third metacarpophalangeal joint which was managed conservatively with splinting. Her first pregnancy was complicated by worsening of her fibroid-related pain in the first trimester and she was commenced on regular analgesia, antiemetics and aspirin (due to concerns of predicted low birth weight). Following delivery she was not on any regular medications. There was no history of drug allergies. Following assessment at the maternity day unit it was concluded that her shoulder tip pain was related to her fibroids and she was discharged with analgesia and safety-netting.

**Figure 1:**
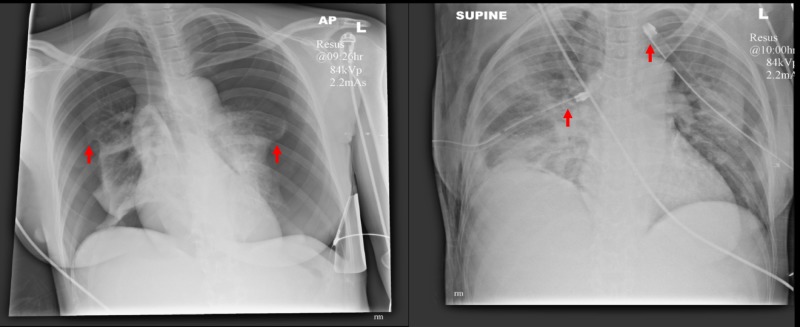
Anteroposterior chest radiographs pre- and post-drain insertion with lung edges and drain tips highlighted.

Two days later, she was admitted as a priority call to our emergency department with a working diagnosis of possible massive pulmonary embolism from the ambulance service. On arrival she was in extremis. Her airway was patent. She was unable to speak with a respiratory rate of 56 and oxygen saturations of 56% on non-rebreather mask (15L of oxygen). Her trachea was central and there was poor air entry bilaterally. She was in shock with a heart rate of 170 beats per minute and unrecordable blood pressure. Point-of-care echocardiography carried out by the emergency department team demonstrated good biventricular systolic function without right ventricular dilatation or pericardial effusion. There was absent pleural sliding bilaterally on thoracic ultrasound. Chest X-ray demonstrated large bilateral pneumothoraces (see Figure 1). Fifty milligrams of intravenous ketamine was given to provide analgosedation for surgical thoracostomies and chest drain insertion. Both pneumothoraces were under tension with audible hiss and rapid physiologic improvement. Subsequent radiograph demonstrated substantial resolution with ex-vacuo pulmonary oedema and new subcutaneous emphysema.

**Figure 2:**
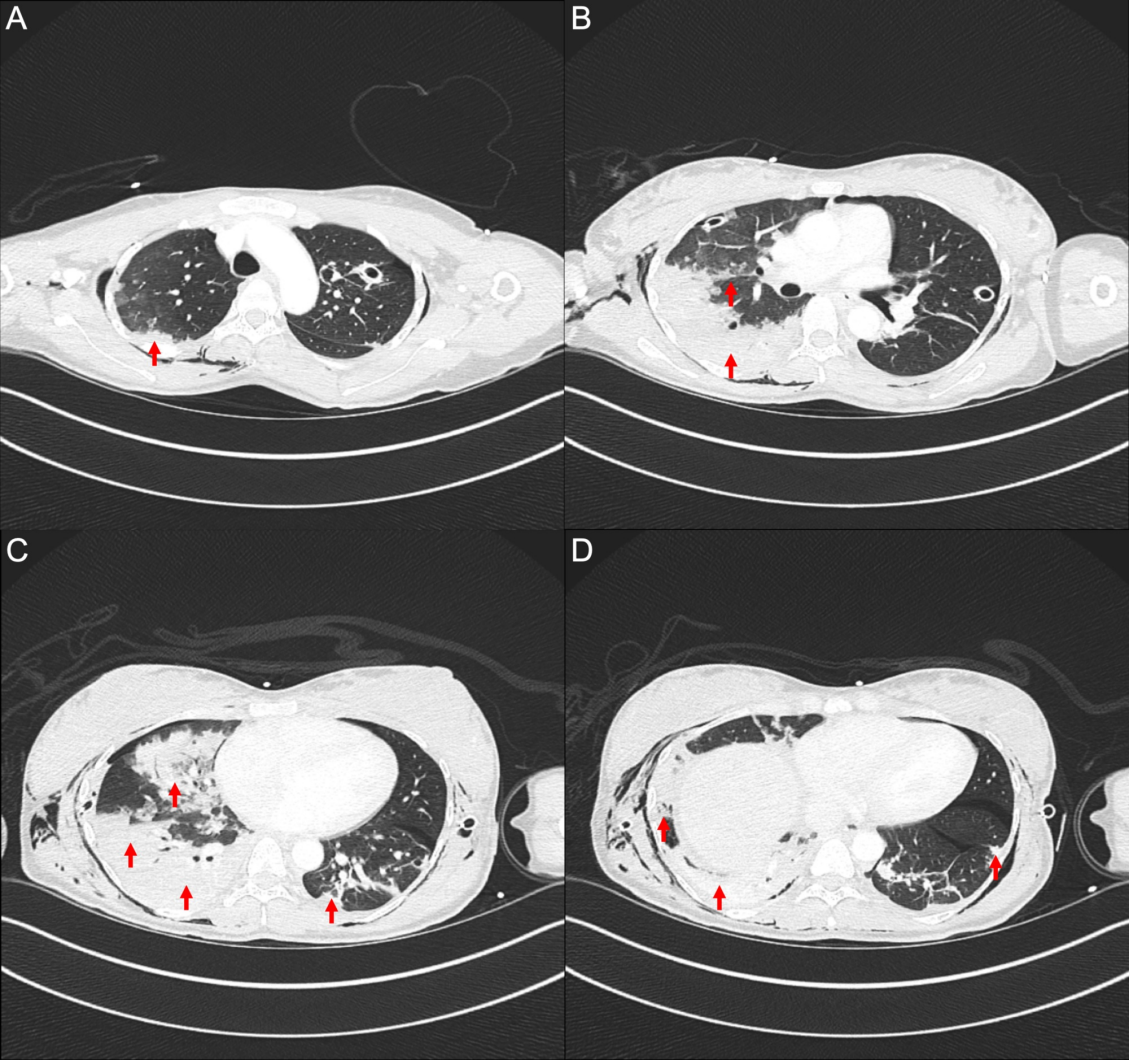
Computed tomography of the chest with iodinated contrast. Selected axial slices from the apices (A) to lung bases (D) demonstrating extensive, predominantly peripheral consolidations and surrounding ground-glass opacities with a crazy paving pattern present throughout the right lung and left lower lobe.

Following stabilisation she underwent computed tomography which additionally demonstrated signs of interstitial lung disease (see Figure 2). There was an 8.3 by 6 cm pedunculated uterine leiomyoma.

She was transferred to a tertiary cardiothoracic centre for further assessment and care. Due to persistent left-sided air leak she underwent unilateral video-assisted thoracoscopic surgery. Apical bullous disease was noted with normal appearances of the horizontal fissure and diaphragm and she underwent left apicectomy and parietal pleurectomy. Histopathological assessment found areas of subpleural fibrosis, patchy interstitial fibrosis and emphysematous changes with very occasional collections of bland spindle-shaped cells at the margins of dilated airspaces. These findings in conjunction with immunohistochemistry (Smooth Muscle Actin and Progesterone Receptor positivity with equivocal to negative Human Melanoma Black 45) suggested an underlying diagnosis of pulmonary lymphangioleiomyomatosis.

Serial imaging demonstrated persistent basal left-sided hydropneumothorax and on the sixth post-operative day she had a 12-French Seldinger chest drain inserted by interventional radiology to good effect. She was discharged with outpatient cardiothoracic surgical and respiratory medicine follow-up to evaluate the need for follow-on right-sided video-assisted thoracic surgery and further assessment of her interstitial lung disease.

## Discussion

To our knowledge this is the first published report of bilateral tension pneumothoraces following labour.

The physiologic changes of normal pregnancy are dramatic. As term approaches, total respiratory compliance falls yet tidal volumes increase to meet the new metabolic demands. Negative intrapleural pressures rise during pregnancy and the functional residual capacity falls [3]. The gravid uterus elevates the intra-abdominal pressure, often falling into the range of intra-abdominal hypertension or even abdominal compartment syndrome [4]. During the second phase of labour the parturient experiences an overwhelming desire to push. Though we were unable to identify published direct measurement of pressure changes during human labour, the usual target for volunteers to maintain in clinical studies of the Valsalva maneuver is 54 cmH20 [5].

Thus labour entails a ‘perfect storm’ for barotrauma to unmask anatomical defects and rarely ensuing unilateral pneumothorax [6], pneumomediastinum (Hamman’s syndrome) [7], pneumopericardium [8], oesophageal perforation [9] and even diaphragmatic rupture [10] have all been described. In this case, signs of underlying pulmonary lymphangioleiomyomatosis were subsequently identified. This rare disease is characterised by proliferation of abnormal smooth muscle-like cells leading to progressive cystic destruction of lung parenchyma, lymphatic changes and intra-abdominal tumours. Lymphangioleiomyomatosis cells express sex hormone receptors and clinical deterioration during pregnancy is recognised [11].

Bilateral tension pneumothoraces pose a clinical conundrum - the trachea is central, auscultation signs may be symmetric yet decisive early intervention is required. A major advance in acute medicine is the increasing adoption of point-of-care ultrasound and this virtually excluded a causative pulmonary embolism or cardiomyopathy in this case. Although the presence of pleural sliding on ultrasound excludes pneumothorax at the segment imaged, its absence is not pathognomonic of pneumothorax (unlike the ‘lung-point sign’) [12].

The immediacy required to relieve a tension pneumothorax, exacerbated by the frequent failure of needle decompression [13], may lead to ineffective local anaesthesia. Ketamine may provide both analgesia and sedation, though dose reductions are warranted in shocked patients despite its relative cardiovascular stability compared to other agents [14]. In the peripartum context, breastfeeding should be avoided for 12 hours after a dose of ketamine and it may impact on neonatal respiration if used for general anaesthesia for caesarean section [15].

## Conclusions

In contemporary UK practice cardio-respiratory and infectious complications are the leading causes of maternal death. Pregnancy and in particular labour may result in catastrophic barotrauma in those with predisposing risk factors. This case highlights the necessity of immediate assessment and intervention followed by thorough investigation for an underlying cause. All clinicians caring for patients in the peripartum period should be aware of these rare but important clinical entities.
